# One-Step Hydrothermal Synthesis of Yellow and Green Emitting Silicon Quantum Dots with Synergistic Effect

**DOI:** 10.3390/nano9030466

**Published:** 2019-03-20

**Authors:** Zhixia Zhang, Chunjin Wei, Wenting Ma, Jun Li, Xincai Xiao, Dan Zhao

**Affiliations:** 1 School of Pharmaceutical Sciences, South-Central University for Nationalities, Wuhan 430074, China; zhixia_z@163.com (Z.Z.); wcj407704@163.com (C.W.); tingwm1993@163.com (W.M.); lijun-pharm@hotmail.com (J.L.); xcxiao@126.com (X.X.); 2 National Demonstration Center for Experimental Ethnopharmacology Education (South-Central University for Nationalities), Wuhan 430065, China

**Keywords:** silicon quantum dots, synthesis, one-pot hydrothermal method, synergistic effect

## Abstract

The concept of synergistic effects has been widely applied in many scientific fields such as in biomedical science and material chemistry, and has further attracted interest in the fields of both synthesis and application of nanomaterials. In this paper, we report the synthesis of long-wavelength emitting silicon quantum dots based on a one-step hydrothermal route with catechol (CC) and sodium citrate (Na-citrate) as a reducing agent pair, and N-[3-(trimethoxysilyl)propyl]ethylenediamine (DAMO) as silicon source. By controlling the reaction time, yellow-emitting silicon quantum dots and green-emitting silicon quantum dots were synthesized with quantum yields (QYs) of 29.4% and 38.3% respectively. The as-prepared silicon quantum dots were characterized by fluorescence (PL) spectrum, UV–visible spectrum, high resolution transmission electron microscope (HRTEM), Fourier transform infrared (FT-IR) spectrometry energy dispersive spectroscopy (EDS), and Zeta potential. With the aid of these methods, this paper further discussed how the optical performance and surface characteristics of the prepared quantum dots (QDs) influence the fluorescence mechanism. Meanwhile, the cell toxicity of the silicon quantum dots was tested by the 3-(4,5-dimethylthiazolyl-2)-2,5-diphenyltetrazolium (MTT) bromide method, and its potential as a fluorescence ink explored. The silicon quantum dots exhibit a red-shift phenomenon in their fluorescence peak due to the participation of the carbonyl group during the synthesis. The high-efficiency and stable photoluminescence of the long-wavelength emitting silicon quantum dots prepared through a synergistic effect is of great value in their future application as novel optical materials in bioimaging, LED, and materials detection.

## 1. Introduction

As a newcomer to nanomaterials, silicon quantum dots (SiQDs) have recently attracted tremendous attention. SiQDs exhibit tunable fluorescence emission properties, chemical stability, favorable biocompatibility, and low toxicity. Thus, they have shown promising potential in a wide range of fields, including lithium-ion batteries [1,2], biological imaging [3,4], and therapy [5,6].

At present, most prepared SiQDs are blue-emitting ones (λ_em_ < 450 nm) [7,8], but the short excitation wavelength (λ_ex_) greatly limits their application in biochemical detection and imaging because of the background fluorescence interference. Therefore, the synthesis of long wavelength SiQDs has become a focus of researchers. Currently, methods for long wavelength emitting SiQDs synthesis are limited, among which the electrochemical method is commonly used. For example, Tu et al. [9] prepared red-emitting SiQDs by etching a p-type silicon wafer in an electrolyte containing HF and methanol. Kang et al. [10] synthesized SiQDs with an emission range from blue to red through a refluxing route after electrolyzing the silicon wafer in an electrolytic cell. Erogbogbo et al. [11] by pyrolyzing silane and etching, finally acquired red, yellow and green-emitting SiQDs. Besides, Kauzlarich et al. [12] acquired orange-emitting SiQDs by reflexing Mg_2_S and adding normal-butyl blocking through a wet chemical method. However, these reported methods are quite complicated with obvious drawbacks such as expensive experimental equipment and high energy costs. Therefore, the development of new, simple, low energy cost, and environment-friendly synthesis methods have aroused immense interest. As a “bottom-to-up” synthesis method for SiQDs, the one-step hydrothermal route is simple in operation, and SiQDs prepared by this method have excellent dispersity without any requirement for further modification before their practical application. So far, most reported SiQDs synthesized though the one-step hydrothermal route emit blue light [7,8,13], and only a few teams have synthesized green-emitting SiQDs by selecting proper reducing agents [14,15,16]. In a recent work, Ma et al. [14] obtained green-emitting SiQDs with (3-Aminopropyl)triethoxysilane (APTES) as silicon source and ascorbic acid as reducing reagent (QYs = 8.2%). Wang et al. [15] acquired green-emitting SiQDs by choosing APTES as silicon source and sodium ascorbate as reducing reagent (QYs = 21%). Han et al. [16], by using DAMO as silicon source and CC as reducing reagent, finally prepared green-emitting SiQDs with QYs at 7.1%. However, the requirement for a large amount of raw materials, the low QYs, and the weak stability of prepared SiQDs turn out to be drawbacks of these syntheses. To overcome the disadvantages, synergistic effects have been introduced to improve the one-step hydrothermal route, to acquire SiQDs with better optical properties, chemical stability and high QYs.

The synergistic effect has been widely applied in the fields of material synthesis [17,18], pharmacology [19,20], and chemistry [21,22], as well as in chemical synthesis, and has been proven to effectively improve the output of the products and simplify the synthesis process. For example, Chen et al. [23] employed the synergistic effect between the metal–organic framework Pd@MOF and metal nanoparticles PdNPs to realize a one-step multiple cascade reaction to synthesize secondary arylamines; Haddleton [24] and his team used a congregation of photo-radicals based on the synergistic effect between CuBr_2_ and tertiary amine, to speed up the synthesis of acrylate and improve the monomer conversion rate, and acquired polyacrylate with excellent uniformity and stability. Moreover, the synergistic effect also shows its effectiveness in the synthesis of fluorescence nanomaterial. The synergistic effect between different elements during the synthesis of N/S [25] or N/P [26] co-doped carbon dots could enhance the optical-chemical activity of prepared carbon dots (CDs). Recently, our team [27] reported a synergistic effect synthesis strategy for preparing SiQDs with super-high QYs (84.92%) by choosing Na-citrate and thiourea as reagent pair. Under optimal excitation environment, the prepared SiQDs emit blue fluorescence (λ_em_ = 452 nm). Therefore, the impact of the synergistic effect of double reducing reagents upon the preparation of long-wavelength-emitting QDs was proven to be effective and positive.

In this study, we report a facile one-step route to prepare stable long-wavelength-emitting SiQDs via the synergistic effect between the reducing reagents CC and Na-citrate. The impacts of different synthesis environment parameters upon the optical properties of prepared SiQDs were explored. It was discovered that the reaction temperature could effectively adjust the emission wavelength (λ_em_) of the product. Besides, we compared the SiQDs synthesized with the reagent pair (CC and Na-citrate) and SiQDs synthesized with only Na-citrate as reagent. The size, surface properties, and elemental composition of these two SiQDs were investigated to reveal their optical characteristics and the emission mechanism. The synergistic effect between the double reducing reagents was proven to be effective in the synthesis of stable long-wavelength-emitting SiQDs with efficient photoluminescence, and is of certain significance in the application as an ideal optical material.

## 2. Materials and Methods 

### 2.1. Reagents and Instruments

Sodium citrate (99.0%) was obtained from Shanghai Zhan Yun Chemical Co., Ltd. (Shanghai, China). N-[3-(Trimethoxysilyl)propyl]ethylenediamine (95.0%) and catechol (≥99.0%) were purchased from Aladdin Chemistry Co., Ltd., (Shanghai, China) Sodium sulfite (≥99.0%), ascorbic acid (≥99.0%), thiourea (≥99.0%), urea (≥99.0%), dimethyl sulfoxide (DMSO) (≥99.5%) and acetonitrile (≥99.0%) were obtained from Sinopharm Chemical Reagent Co., Ltd. (Shanghai, China). MTT (≥98.0%) was purchansed form Sigma-Aldrich Co., Ltd. (Merck KGaA, Darmstadt, Germany). All solutions were prepared using Milli-Q water (Millipore, Burlington, MA, USA) as the solvent. 

UV–visible absorption spectra were acquired with a Lambda-35 UV-visible spectrophotometer (PerkinElmer Company, Waltham, MA, USA) to determine the bandgap absorption of SiQDs. Fluorescence spectra were recorded on a LS55 spectrofluorometer (PerkinElmer Company). HRTEM images were obtained with a JEM2100F transmission electron microscope (Japan Electron Optics Laboratory Company, Tokyo, Japan) and EDS data were obtained by its annex. FT-IR spectra were obtained on a Nicolet 6700 spectrometer (Thermo Fisher Scientific, Waltham, MA, USA). Zeta-potential measurement was carried out on a Zetasizer nanoseries ZEN3690 (Malvern, UK). The relative QYs of as-prepared SiQDs were measured according to the literature with Rhodamine 6G in ethanol (QY = 95%) as a reference standard. All optical measurements were performed at room temperature under ambient conditions.

### 2.2. Preparation of Silicon QDs

Na-citrate (9.24 × 10^−5^ mol·L^−1^) was dissolved in water (10 mL) under stirring for 10 min. Then, DAMO (0.2 mL) was added to the solution and stirring continued for 10 min. Finally, 1.1 mg CC was added to the solution with stirring for 1 min. Subsequently, the resulting products were heated in a Teflon-equipped stainless-steel autoclave at 130 °C or 150 °C for 5 h. After naturally cooling to room temperature, four volumes of acetonitrile were added to the obtained solution and the mixture was centrifuged at 8000 rpm for 15 min to remove raw material.

The same concentration of sodium sulfite, ascorbic acid, thiourea, or urea was used instead of Na-citrate, and heated at 130 °C for 5 h to obtain SiQDs of different reducing agent combinations to compare their relative quantum yields. The relative PLQYs of as-prepared SiQDs were measured according to the literature with Rhodamine 6G in ethanol (QY = 95%) as a reference standard.

### 2.3. MTT Method

The MTT method was used to detect the cell viability of two kinds of SiQDs. The L02 cells were seeded at a density of 1 × 10^4^ cells per well with 100 µL of culture medium in 96-well plates and placed for cell growth for 24 h in a 37 °C, 5% CO_2_ humidified incubator. The appropriate amount of yellow-emitting SiQDs (y-SiQDs) and green-emitting SiQDs (g-SiQDs) were dissolved in ultrapure water and added to Dulbecco’s modified eagle medium (DMEM) to a concentration of 200 μg/mL. Different concentrations of dilution samples were added per well (The concentration of DMSO was less than 1‰). After adding 100 μL of MTT solution to each well, it was placed in the incubator for 30 min. Its supernatant was discarded after culturing the cells for 24 h, and then 150 μL DMSO per well was added. After shaking in the dark for 10 min, the microplate reader detected the optical density value (OD) at 562 nm. The cell survival rate is calculated as follows. IC50 values were calculated by GraphPad Prism 6 (GraphPad Software, Inc., San Diego, CA, USA).

$$\mathrm{Cell}\;\mathrm{viability}\left(\%\right)=\frac{\mathrm{OD}\;\mathrm{value}\;\mathrm{of}\;\mathrm{experiment}\;\mathrm{group}-\mathrm{OD}\;\mathrm{value}\;\mathrm{of}\;\mathrm{control}\;\mathrm{group}}{\mathrm{OD}\;\mathrm{value}\;\mathrm{of}\;\mathrm{negative}\;\mathrm{control}\;\mathrm{group}-\mathrm{OD}\;\mathrm{value}\;\mathrm{of}\;\mathrm{control}\;\mathrm{group}}$$

## 3. Results and Discussion

### 3.1. The Optimization of the Synthesis Environment for SiQDs

#### 3.1.1. Filtration of the Proper Selection of Reducing Reagents for SiQDs Preparation

The reducing reagent is one of the most important factors of the synthesis that determines the optical properties of the prepared SiQDs. Proper selection of reducing reagents can effectively change the fluorescence intensity and λ_em_ of the products. Based on the published literature and our previous work, it was discovered that ascorbic acid (VitC) [14], sodium ascorbate (VitC-Na) [15], and CC [16,27] are the ideal candidates for the preparation of long-wavelength emitting SiQDs. The preparation process with one of them as reducing reagent could produce green-emitting SiQDs (λ_em_ ~ 520 nm), but the QYs of the production were quite low (<5%). Adjusting the traditional synthesis environment, such as the adjustment of reactant ratio, reaction time, and temperature, could not further improve the optical properties of the product. Therefore, this paper reports the introduction of a second reducing reagent into the synthesis process, to improve the stability and QYs of the products through a synergistic effect. 

Since the synergistic effect between double reducing reagents has been proven to be effective in improving the optical properties of blue-emitting SiQDs [27], the basic reducing reagent should first be selected out of the above-mentioned three candidates (VitC, VitC-Na, and CC). Based on that, the second reducing reagent should be selected to combine with the basic reagent to improve the optical and chemical properties of the SiQDs. As is known, Na-citrate is an excellent reducing reagent [28]. The prepared SiQDs exhibit QYs as high as 73.3% with DAMO as silicon source and Na-citrate as reducing reagent. Na-citrate was thus chosen as one candidate of the reducing reagent pair. Then, three other reducing reagent candidates were matched with Na-citrate to test their ability for improvement. With the reactant ratio fixed at n(DAMO):n(CC):n(Na-Citrate) = 1:0.11:0.47 (cDAMO = 8.29 × 10^−2^ mol·L^−1^), reaction temperature at 130 °C, and reaction time for 5 h, the prepared SiQDs emitted yellow fluorescence with Na-citrate/CC as reducing reagent pair. They also maintained excellent optical properties and chemical stability with QYs as high as 29.4%. Na-citrate/VitC and Na-citrate/VitC-Na pairs could be used to prepare green-emitting SiQDs (λ_em_ ~ 500 nm) with QYs of 5.5% and 6.4%. Comparing with the results of the three groups, CC was selected as one of the reducing reagents for the synergistic effect.

**Figure 1:**
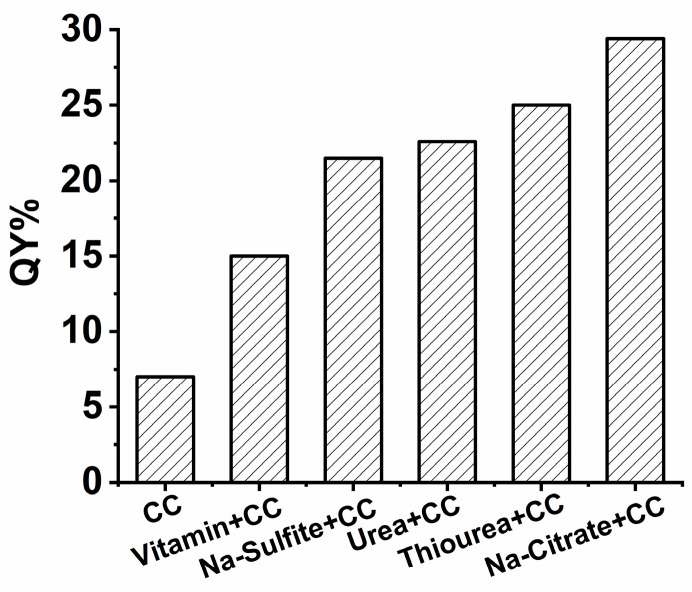
QYs of SiQDs synthesized with single reductant (CC) and double reductants (CC with other reductants).

With CC chosen as one candidate, the other reducing reagent of the pair was changed to assess more pairing possibilities. In the synthesis process, the inorganic reducing reagent sodium sulfite, organic reducing reagents thiourea, urea, and VitC have all been proven to have excellent reducing abilities. As shown in Figure 1, the combinations of CC and any one of these reducing reagents also resulted in acquiring long-wavelength emitting SiQDs, with their QYs all higher than 15%, obviously higher than that of the SiQDs prepared by the single reducing reagent CC. This proves that the synergistic effect of double reducing reagents could effectively improve the optical properties of SiQDs. This might be attributable to the strong reducing abilities of Na-citrate, as well as the unique structure of CC which is beneficial for the red-shift of λ_em_ of the products. More detailed discussion on the synthesis mechanism is presented in the later part of this paper.

#### 3.1.2. The Impact of Synthesis Parameters upon the Optical Properties of SiQDs

**Figure 2:**
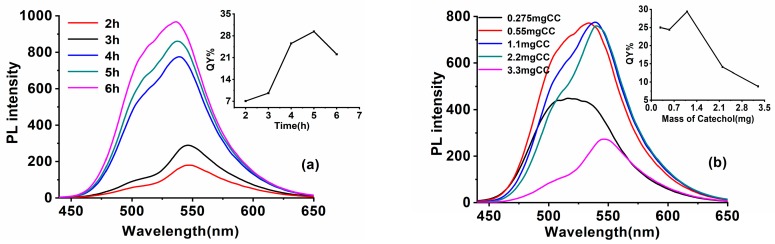
PL spectra of SiQDs synthesized with CC and Na-Citrate (**a**) with different addition mass of Na-citrate (**b**) with different addition masses of CC. The insets show the corresponding QYs.

The ratio of reactants, reaction temperature, and time are the key factors for SiQDs synthesis. Figure 2a shows the impact of the addition mass of Na-citrate. Fixing the reaction materials ratio of c(DAMO):c(CC) at 1:0.11, the solution was heated at 130 °C for 5 h. When the addition mass of Na-citrate was raised to 13.6 mg, the fluorescence intensity of the prepared SiQDs reached a maximum. However, the changed amounts of Na-citrate did not change the λ_em_ of the prepared SiQDs. Changing the addition amount of CC, on the other hand, had an impact upon the λ_em_. As shown in Figure 2b, with other synthesis parameters fixed, and n(DAMO):n(Na-Citrate) set at 1:0.47, the λ_em_ of SiQDs redshifts from 518 nm to 546 nm with increasing CC amount. The QYs of the prepared SiQDs reach their maximum at 29.4% with the amount of CC rising to 1.1 mg. 

**Figure 3:**
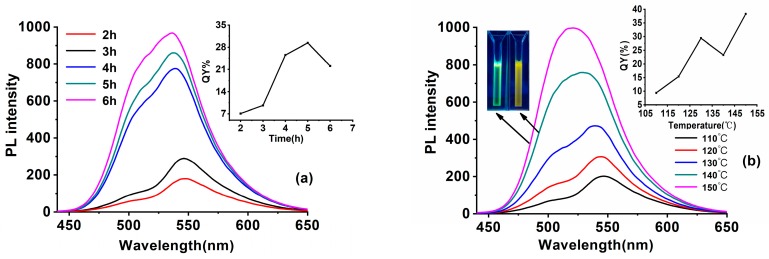
PL spectra of SiQDs synthesized with CC and Na-citrate at (**a**) different reaction time and (**b**) at different reaction temperature. Inset: the picture shows the increasing trend of QYs and the photos are of g-SiQDs and y-SiQDs.

On fixing the other synthesis parameters, the increased reaction time from 2 h to 6 h enhanced the fluorescence intensity of the prepared SiQDs, with the λ_em_ blue shifts from 546 nm to 535 nm (Figure 3a). The QYs of SiQDs reach their maximum with a reaction time of 5 h. 

Reaction temperature is also one of the important factors affecting the optical properties of SiQDs. Figure 3b displays the different regular patterns of the QDs syntheses: the drop of reaction temperature from 150 °C to 110 °C renders λ_em_ red shifts from 520 nm to 545 nm. A significant change in the color of SiQDs from green to orange could be observed under UV light. Surface chemistry is the key factor that determines the λ_em_ change of the prepared SiQDs [29]. The long-time exposure in the high-temperature oxidation environment would destroy the structure of CC, preventing it from forming luminophores on the surface of the SiQDs while the silane mostly combines with Na-citrate, leading to short-wavelength emitting SiQDs. On the other hand, the low temperature environment would greatly decrease the fluorescence intensity. The QYs of prepared SiQDs dropped from 39.3% to 9.4% when the reaction temperature dropped from 150 °C to 110 °C. That may because the low temperature is not beneficial to the decomposition of silane to prevent the surface defects of the SiQDs.

Therefore, through a series of experiments, the best synthesis environment for high QYs yellow-emitting SiQDs (y-SiQDs) and green-emitting SiQDs (g-SiQDs) were acquired. As shown in the insert of Figure 3b, with n(DAMO):n(CC):n(Na-Citrate) fixed at 1:0.11:0.47, reaction time at 5 h, y-SiQDs were acquired at a temperature of 130 °C with QYs at 29.4%, and g-SiQDs were acquired at 150 °C with QYs at 38.3%. Though both were prepared with the same raw materials, these two SiQDs exhibited different optical properties. The fluorescence emission peak of y-SiQDs (Figure S1) is obviously asymmetric, and two obvious UV absorption peaks can be observed at 233 and 254 nm, while the g-SiQDs (Figure S2) does not exhibit a similar spectra shape.

### 3.2. Characterization of SiQDs and Mechanism Discussion

#### 3.2.1. Three Dimensional Fluorescence Spectra

The three-dimensional fluorescence spectra could directly describe the changes of λ_em_ and fluorescence intensity with the λ_ex_. It was thus used to compare the optical property differences of the b-SiQDs (DAMO as silicon source, Na-citrate as reducing agent), SiQDs(CC) (DAMO as silicon source, CC as reducing agent) y-SiQDs, and g-SiQDs.

**Figure 4:**
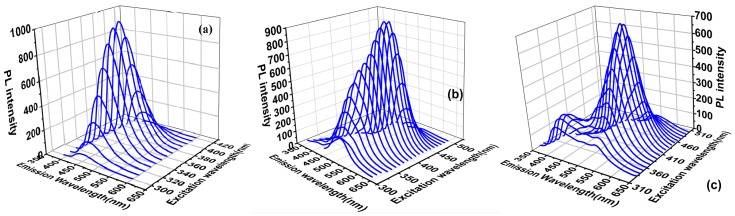
Three-dimensional fluorescence spectra of the three kinds of (**a**) b-SiQDs; (**b**) g-SiQDs, and (**c**) y-SiQDs.

Figure 4a shows the change of the b-SiQDs fluorescence emission spectra with λ_ex_ in the range of 300–420 nm (gap at 10 nm). With the increase of λ_ex_, the fluorescence intensity continues to increase and reaches a maximum at λ_ex_ = 370 nm. Further increase of λ_ex_ leads to a rapid decrease of fluorescence intensity, with its λ_em_ staying at about 453 nm. 

Figure S3 shows the three-dimensional fluorescence spectra comparing the three-dimensional spectra of SiQDs(CC). We can find that, the λ_em_ of SiQDs(CC) only changes in fluorescence intensity at 550 nm with the λ_ex_ of SiQDs(CC) increasing from 310 nm to 500 nm. Also there is no occurrence of emission peak shift, which is similar to the cas of the three-dimensional spectra of b-SiQDs.

Figure 4b exhibits a change of g-SiQDs fluorescence emission spectra with λ_ex_ in the range of 310–500 nm (gap at 10 nm). The emission peak of g-SiQDs exhibits slight blue-shift when λ_ex_ increases in the range of 310–360 nm, with gradual increase of fluorescence intensity. Further increase of λ_ex_ (360–420 nm) makes the emission peak redshift with further increase of fluorescence intensity, while the fluorescence intensity reaches the maximum (λ_em_ = 527 nm) at λ_ex_ = 420 nm. When λ_ex_ reaches the range of 430–500 nm, the redshifts of the emission peak continue with decrease of fluorescence intensity. 

However, the change of y-SiQDs in their three-dimensional fluorescence spectra is more complicated than the other two. Figure 4c displays the change of the y-SiQDs fluorescence emission spectra with λ_ex_ in the range of 330–550 nm (gap at 10 nm), with two different changes in behavior being observed. When λ_ex_ is in the range of 330–370 nm, two emission peaks exist at 395 nm and 540 nm. With the increase of λ_ex_, the fluorescence intensity at 395 nm decreases, while the one at 540 nm enhances. When λ_ex_ rises up to the range of 380 to 500 nm, the emission peak at 395 nm disappears, and the fluorescence intensity of y-SiQDs increases with λ_em_. The intensity reaches a maximum at λ_ex_ = 420 nm. Further increase of λ_em_ would lead to redshift of the emission peak and decrease of fluorescence intensity.

Therefore, the particle size distribution results of y-SiQDs were used to demonstrate the possibility of the presence of two sizes of nanoparticles in y-SiQDs. As shown in Figure S4a, when performing the particle size distribution statistics of y-SiQDs, the number of particles around ~5 nm is also higher than the normal level, in addition to the large particles of y-SiQDs. This shows that the complex behavior of the 3D spectra of y-SiQDs is likely to originate from the presence of two types of SiQDs in the sample. This phenomenon may be due to a competitive reaction between the two reducing agents and the silicon source during the synthesis. Na-citrate is an excellent reducing agent that is more easily combined with silane to produce blue-emitting SiQDs. 

#### 3.2.2. HRTEM Imaging

**Figure 5:**
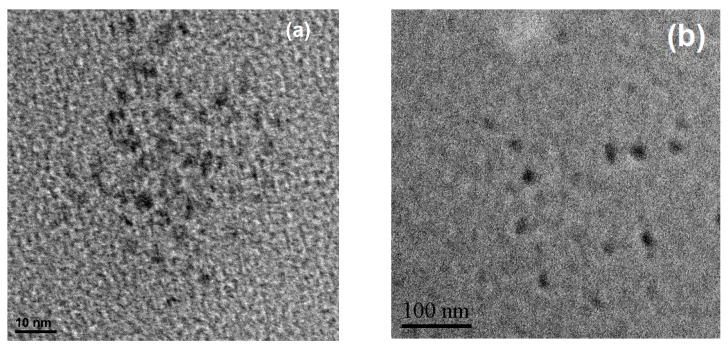
The TEM images of (**a**) b-SiQDs; (**b**) y-SiQDs.

The particle size of SiQDs is one of the crucial factors for its optical properties. Thus, the transmission electron microscope was applied to study the particle size and morphology of the prepared b-SiQDs and y-SiQDs. Both SiQDs exhibited uniform distribution and spherical morphology (Figure 5). The diameter distribution of the b-SiQDs is in the range of 2–3.5 nm with average diameter at 2.8 nm; while the diameter distribution of y-SiQDs is in the range of 10–13 nm with average diameter at 12 nm. Figures S4 and S5 shows the particle size distribution histograms and the lattice plane of two kinds of SiQDs corresponds to the d-spacing of the cubic diamond structure of silicon giving the (111) plane with 0.31 nm spacing. It is thus proven that the increase of particle size could effectively make the emission peak redshift.

#### 3.2.3. FT-IR Spectrometer

**Figure 6:**
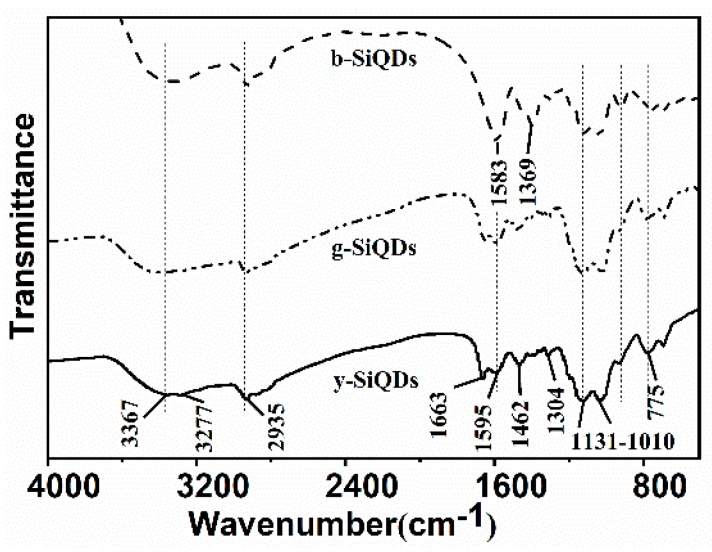
FT-IR spectrum of the three kinds of SiQDs (b-SiQDs, g-SiQDs, and y-SiQDs).

A FT-IR spectrometer was used to examine the functional groups on the surface of SiQDs. As shown in Figure 6, the surface functional groups of b-SiQDs, G-SiQDs, and y-SiQDs are quite similar, including the stretching vibration of N–H [30], O–H [31] at 3277 cm^−1^ and 3367 cm^−1^, and the stretching vibrations of –CH=N– [32] at 1595 cm^−1^. Stretching and bending vibrations due to methyl groups [33] were represented by the bands at 2935 cm^−1^ and 1462 cm^−1^, the asymmetrical deformation vibration of Si–O and the stretching vibrations of Si–O–Si at 1310 cm^−1^ and 1130–1010 cm^−1^ [27]. These results show that the existence of hydroxyl and ammonia groups could effectively enhance the water-solubility and stability of the prepared SiQDs. However, the spectra of b-SiQDs do not exhibit carbonyl groups [34] absorption peak at 1663 cm^−1^ as is the case with both y-SiQDs and g-SiQDs. The low vibration frequency of the carbonyl groups proves the presence of large conjugation systems on both sides of the carbonyl groups, leading to a weakened strength of the double bond. The enlarged conjugation system makes the λ_em_ redshift. The zeta potential of y-SiQDs is −17 mV, showing the surface of the SiQDs is negatively charged with the existence of hydroxyl group on the surface.

#### 3.2.4. EDS Spectrum

**Figure 7:**
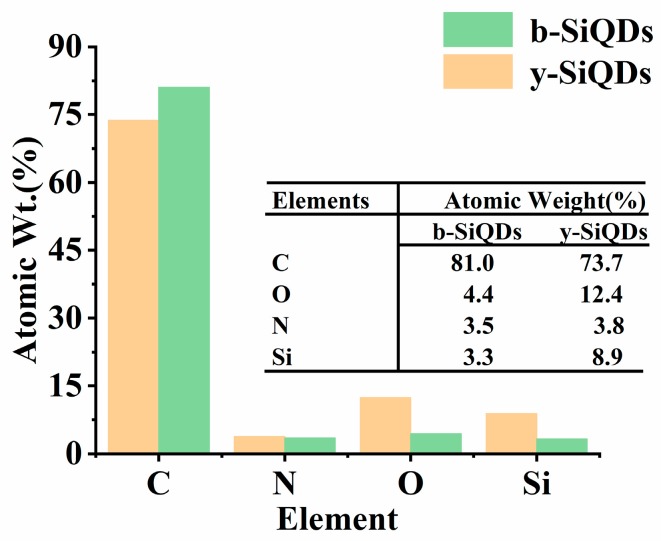
Atomic weight composition (%) of C, N, O, and Si for b-SiQDs and y-SiQDs examined by EDS.

The EDS spectrum was used to analyze the chemical constitutions of y-SiQDs and b-SiQDs (Figures S6 and S7). The contents of C, N, O, and Si elements in both SiQDs were measured, and their relative atomic weight ratios are listed in Figure 7. The relative atomic weight ratios of C to O of b-SiQDs and y-SiQDs are 1:0.05 and 1:0.168, respectively, showing a richer existence of O elements on the surface of y-SiQDs and thus a relative higher oxidation degree. This also proves the existence of carbonyl groups on the surface as shown from the FT-IR spectra of the y-SiQDs.

#### 3.2.5. Study on the Synthesis Mechanism

In our previous work [27], the QYs of SiQDs (with DAMO as the silicon source, sodium oxalate, and citric acid as the reducing agent pair) were effectively improved by synergistic effects, but the λ_em_ did not change. In this paper, we selected the right pair of reducing agents (CC and Na-citrate), which not only improved the QYs of SiQDs, but also caused different degrees of red shift in the emission wavelengths. 

**Figure 8:**
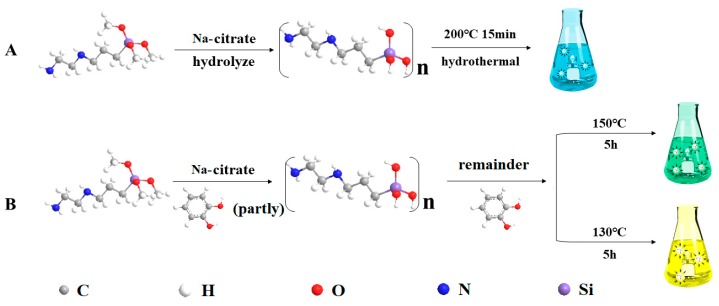
Schematic illustration of the synthesis of (**A**) b-SiQDs and (**B**) g-SiQDs and y-SiQDs.

Figure 8 exhibits the synthesis mechanism of b-SiQDs, g-SiQDs, and y-SiQDs. When we use DAMO as the silicon source and Na-citrate as the reducing agent, the silane DAMO undergoes hydrolysis–reduction–polymerization to form b-SiQDs [13]; when we use DAMO as the silicon source, Na-citrate and CC as the double reducing agent, y-SiQDs are synthesized. As a common excellent reducing agent, Na-citrate is beneficial for the rapid hydrolysis and reduction of silane to form a silicon core. In addition to participating in the reduction of silane during the synthesis, the phenolic hydroxyl group on the surface of the CC can be further combined with the residue on the surface of the hydrolyzed product of DAMO to form a fluorophore having a larger conjugated system and a C=O structure on the surface, which is advantageous for the SiQDs to form long wavelengths and higher QYs. The presence of carbonyl groups on the surface of SiQDs was also confirmed by the higher O/C ratio of EDS observed in the IR spectra.

It is worth noticing that lowering of either reaction temperature or time would make the emission wavelength of the prepared SiQDs redshift. This is different from the synthesis mechanism of other SiQDs. With the same reactants ratio and reaction time (5 h), y-SiQDs can be acquired at a temperature of 130 °C, while higher temperature (150 °C) would lead to the formation of g-SiQDs. We suspect that the possible reason is that this is not conducive to the stable existence of the CC structure at high temperature conditions, and thus cannot participate in the formation of the surface luminescent groups of y-SiQDs.

### 3.3. Application of the Prepared SiQDs

#### 3.3.1. Optical Stability of the SiQDs

The optical stability is one of the most crucial properties of QDs for their wide application in the fields of analytical detection, optical sensing, and nonlinear optical materials. The optical stability of prepared SiQDs was tested by setting it under natural light and measuring its fluorescence intensity every five minutes. The fluorescence intensities of both y-SiQDs and g-SiQDs exhibit no obvious decrease on 30 min of exposure to natural light (Figure S8), proving their excellent optical stability.

#### 3.3.2. Cytotoxicity of the SiQDs

**Figure 9:**
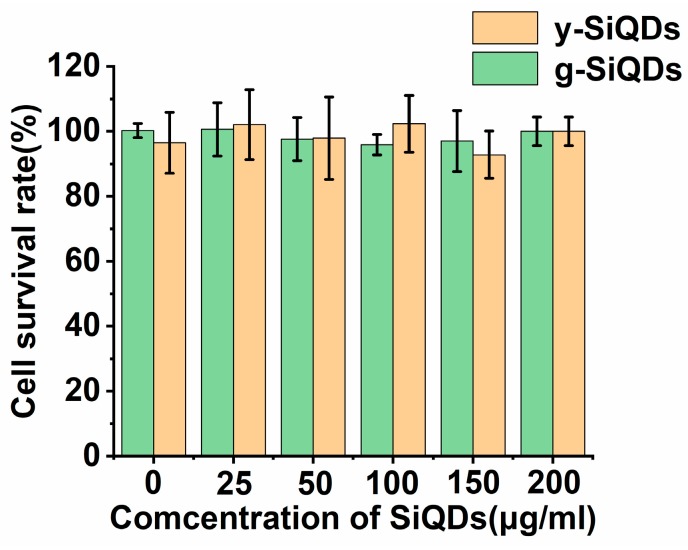
Cell viability (%) measured by MTT assay. The L02 cells were incubated with the SiQDs for 24 h at 37 °C. All results were presented as the mean ± standard deviation (SD) from three independent experiments with four wells in each.

The cytotoxicity of SiQDs is also an important factor with regard to their final application in an organism [35,36]. The MTT method is the common way to assess the cytotoxicity of QDs. y-SiQDs, and g-SiQDs in different concentrations were used to incubate the L02 cell for 24 h, and then the activity of the cells was measured. Figure 9 shows that two kinds of SiQDs exhibit really low cytotoxicity with almost no interference to the growth of the cells. The excellent stability and safety could guarantee their potential application as probes in biochemical fields. 

#### 3.3.3. Fluorescence Ink

**Figure 10:**
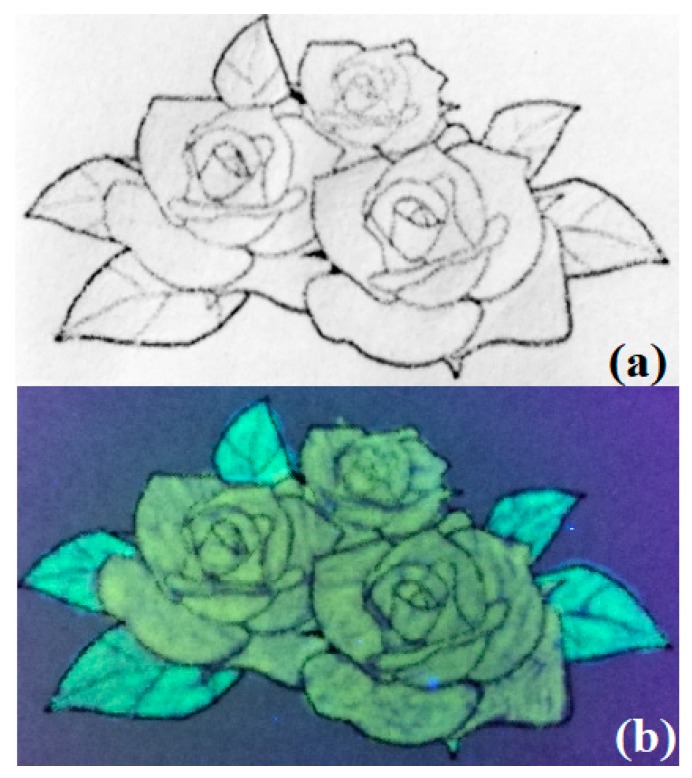
Photos of pictures painted by y-SiQDs and g-SiQDs. Photo (**a**) was photographed under daylight and (**b**) was photographed under UV-light (365 nm).

Since y-SiQDs and g-SiQDs exhibit excellent optical stability and low toxicity at high concentration, they show great possibility as new biocompatible fluorescence inks. Figure 10a,b shows the pattern drawn with y-SiQDs and g-SiQDs on filter paper under natural light and UV light (365 nm). The filter paper exhibits a strong bright yellow and green fluorescence pattern under UV light. This proves that y-SiQDs and g-SiQDs could be used as a fluorescence invisible ink, or further be applied in the field of banknote anti-counterfeiting techniques [37].

## 4. Conclusions

This paper reports the preparation of stable y-SiQDs and G-SiQDs through the one-step hydrothermal route with synergistic effects between double reducing reagents CC and Na-citrate, with the silane coupling agent N-[3-(trimethoxysilyl)propyl]ethylenediamine (DAMO) as silicon source. The QYs of the prepared y-SiQDs and g-SiQDs reach as high as 29.4% and 38.3%. The optical properties of both SiQDs were characterized and tested by three-dimensional fluorescence spectra, IR spectra, EDS, and TEM, with further comparison to the b-SiQDs prepared by DAMO and Na-citrate. It was discovered that the participation of the carbonyl group during the synthesis makes the λ_em_ redshift. The preparation of long-wavelength emitting SiQDs through a synergistic effect and their highly efficient and stable photoluminescence ensure their promising application as optical materials.

## Supplementary Materials

The following are available online at https://www.mdpi.com/2079-4991/9/3/466/s1, Figure S1: UV–Vis adsorption and photoluminescence emission spectra of y-SiQDs; Figure S2: UV–Vis adsorption and photoluminescence emission spectra of g-SiQDs; Figure S3: Three-dimensional fluorescence spectra of three kinds of SiQDs(CC); Figure S4: (a) particle size distribution histograms and (b) lattice image y-SiQDs; Figure S5: (a) particle size distribution histograms and (b) lattice image b-SiQDs; Figure S6: EDS spectra of y-SiQDs; Figure S7: EDS spectra of b-SiQDs; Figure S8: The relationship of replaced time with the fluorescence intensity of y-SiQDs and g-SiQDs.
