# Supplementary material for: One-Step Hydrothermal Synthesis of Yellow and Green Emitting Silicon Quantum Dots with Synergistic Effect
Source: Nanomaterials (Basel). 2019 Mar 20;9(3):466. doi: 10.3390/nano9030466 (PMC6474109; doi:10.3390/nano9030466)
Supplement: Supplementary file 1 [file nanomaterials-09-00466-s001.pdf]

Supporting Information

One-Step Hydrothermal Synthesis of Yellow and Green Emitting Silicon Quantum Dots with Synergistic Effect

Zhixia Zhang [1,2], Chunjin Wei [1,2], Wenting Ma [1,2], Jun Li [1,2], Xincai Xiao [1,2] and Dan Zhao [1,2,*]

1 School of Pharmaceutical Sciences, South-Central University for Nationalities, Wuhan 430074, China; zhixia_z@163.com (Z.Z.); wcj407704@163.com (C.W.); tingwm1993@163.com (W.M.); xcxiao@126.com (X.X.); lijun-pharm@hotmail.com (J.L.)

2 National Demonstration Center for Experimental Ethnopharmacology Education (South-Central University for Nationalities), Wuhan 430065, China

* Correspondence: wqzhdpai@163.com; Tel.: +86-1806-208-4690

Figure S1 shows the fluorescence and UV-Vis spectra of y-SiQDs. When the excitation wavelength is 420 nm, the emission wavelength of y-SiQDs is 538 nm. The main absorption peak of ultraviolet light is located at 438 nm. The absorption peak at 233 nm in the UV-Vis spectrum due to π-π* transition of C=C bond.

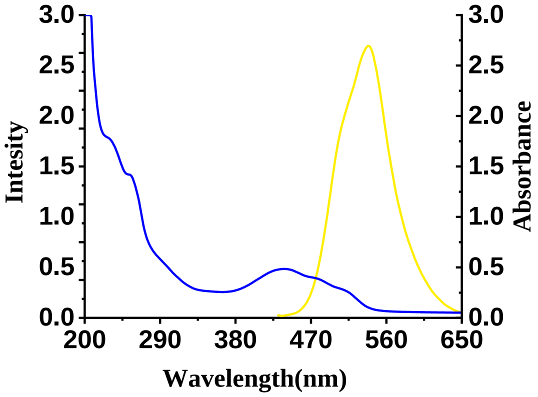

Figure S1. UV-Vis adsorption and photoluminescence emission spectra of y-SiQDs.

Figure S2 shows the fluorescence and UV-Vis spectra of g-SiQDs. When the excitation wavelength is 420 nm, the emission wavelength of y-SiQDs is 520 nm. The main absorption peak of ultraviolet light is located at 430 nm.

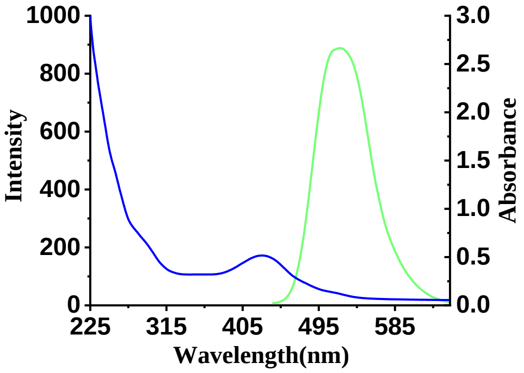

Figure S2. UV-Vis adsorption and photoluminescence emission spectra of g-SiQDs.

Figure S3 shows three-dimensional fluorescence spectra comparing the three-dimensional spectra of SiQDs (CC).

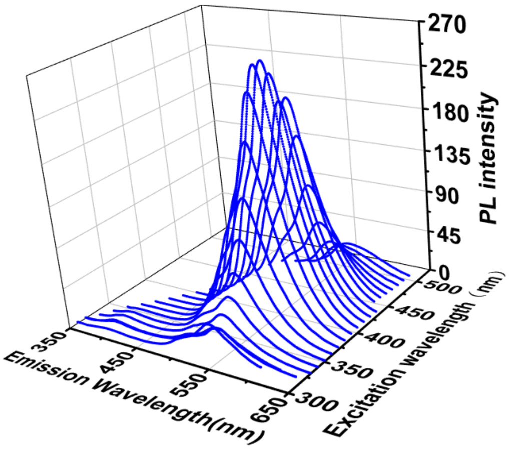

Figure S3. Three-dimensional fluorescence spectra of three kinds of SiQDs(CC).

Figures S4 and S5 Demonstrate the particle size distribution and lattice image of y-SiQDs and b-SiQDs.

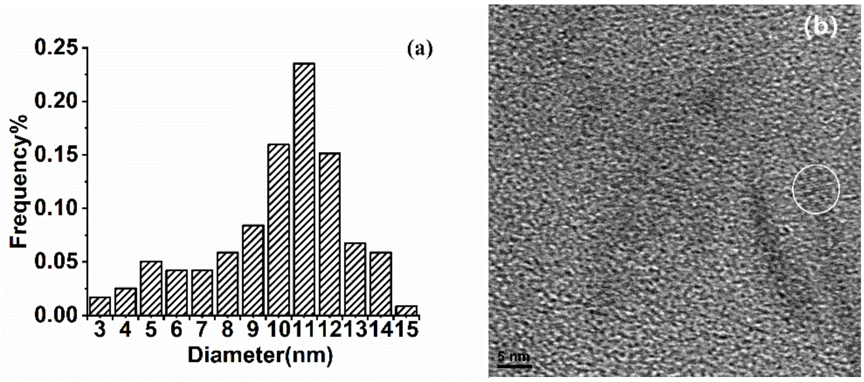

Figure S4. (**a**) particle size distribution histograms and (**b**) lattice image y-SiQDs.

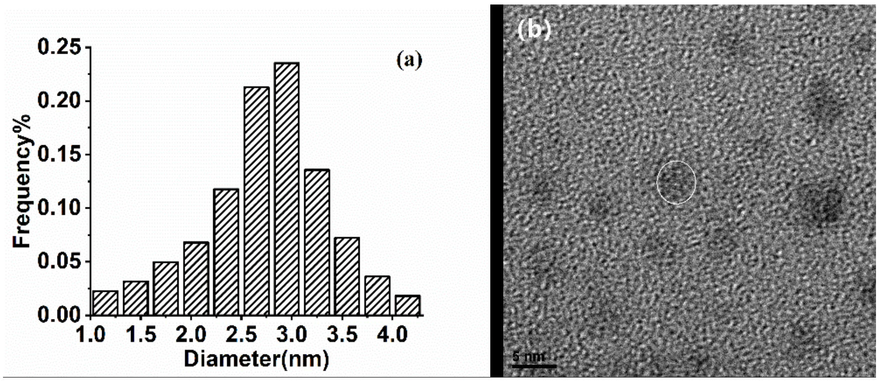

Figure S5. (**a**) particle size distribution histograms and (**b**) lattice image b-SiQDs.

Figure S6 and Figure S7 show the EDS spectra of y-SiQDs and b-SiQDs, respectively. The elemental content of the two SiQDs can be compared though the EDS spectrum.

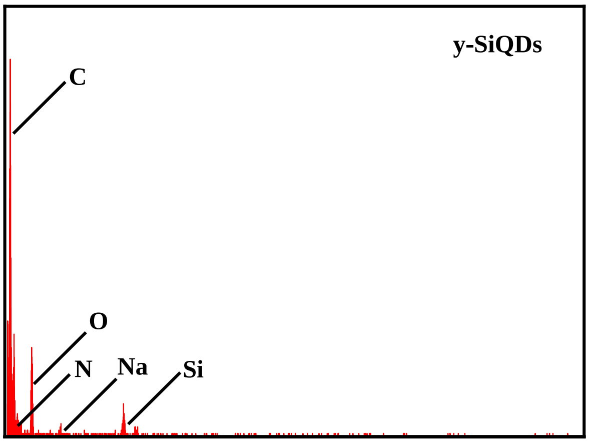

Figure S6. EDS spectra of y-SiQDs.

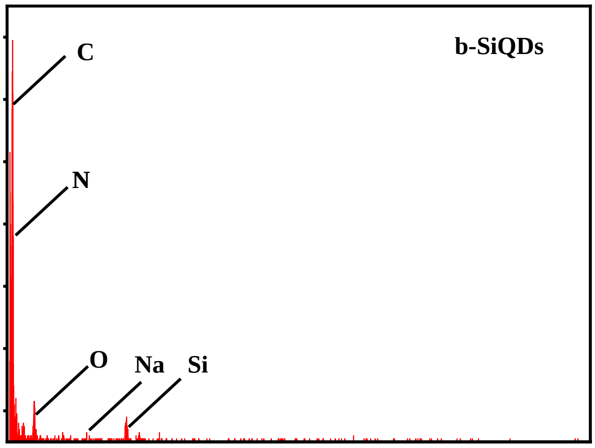

Figure S7. EDS spectra of b-SiQDs.

Figure S8 shows the fluorescence changes of y-SiQDs and g-SiQDs within 30 min of placement. Both two kinds of SiQDs exhibit good light stability.

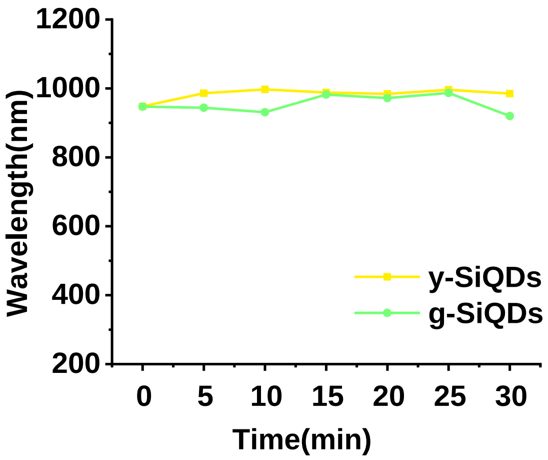

Figure S8. The relationship of replaced time with the fluorescence intensity of y-SiQDs and g-SiQDs.
